# Rapid online assessment of reading and phonological awareness (ROAR-PA)

**DOI:** 10.1038/s41598-024-60834-9

**Published:** 2024-05-04

**Authors:** Liesbeth Gijbels, Amy Burkhardt, Wanjing Anya Ma, Jason D. Yeatman

**Affiliations:** 1 Department of Speech and Hearing Sciences, University of Washington, Seattle, WA USA; 2 Institute for Learning and Brain Sciences, University of Washington, Seattle, WA USA; 3 Division of Developmental-Behavioral Pediatrics, Stanford University School of Medicine, Stanford, CA USA; 4 Stanford University Graduate School of Education, Stanford, CA USA; 5 Stanford University Department of Psychology, Stanford, CA USA; 6 Center for Educational Research at Stanford, 520 Galvez Mall, Stanford, CA 94305 USA; 7 Present Address: Natural Language Applications at Cambium Assessment, Washington, USA

**Keywords:** Phonological awareness, Reading, Literacy, Measurement, Assessment, Dyslexia, Screener, Human behaviour, Sensory processing

## Abstract

Phonological awareness (PA) is at the foundation of reading development: PA is introduced before formal reading instruction, predicts reading development, is a target for early intervention, and is a core mechanism in dyslexia. Conventional approaches to assessing PA are time-consuming and resource intensive: assessments are individually administered and scoring verbal responses is challenging and subjective. Therefore, we introduce a rapid, automated, online measure of PA—The Rapid Online Assessment of Reading—Phonological Awareness—that can be implemented at scale without a test administrator. We explored whether this gamified, online task is an accurate and reliable measure of PA and predicts reading development. We found high correlations with standardized measures of PA (CTOPP-2, r = .80) for children from Pre-K through fourth grade and exceptional reliability (α = .96). Validation in 50 first and second grade classrooms showed reliable implementation in a public school setting with predictive value of future reading development.

## Introduction

Literacy is critical to educational success. Individual differences in reading skills are predictive of educational outcomes throughout schooling and have been linked to socioeconomic and health disparities^1,2^. Although the focus on learning to read begins in kindergarten, there are large individual differences in reading abilities that persist throughout adulthood. Understanding the mechanisms underlying these individual differences is a continuing topic of debate^3–11^. However, there is a near consensus on the importance of foundational skills like phonological awareness for reading development^12–15^. Phonological awareness (PA), or phonological processing more broadly, refers to a metalinguistic skill that enables an individual to reflect upon and manipulate the sound structure of spoken language^16^, at the level of (1) words and syllables, (2) onset and rimes, or (3) phonemes^9,17^, independent of their meaning. PA skills emerge early, develop rapidly throughout childhood^18^, and have important implications for literacy achievement^19^. To learn how to read a child needs to be aware of the arbitrary and conventional correspondence between the sound structure of language and the rules for how it is written^20^.

Important questions remain regarding the relationship between PA and reading development. Are some phonological skills are more closely related to reading development than others (i.e., *syllabic awareness*:^21^, *intrasyllabic awareness*:^22^, *phonemic awareness:*^23^, and *prosodic awareness*:^24^? Do all aspects of PA reflect the same latent construct^25,26^? And does the relationship between different aspects of PA change throughout development? These questions in combination with the reciprocal nature of PA and reading development^27^ have practical implications for reading instruction regarding the importance of distinguishing between different aspects of PA in relation to learning to read (for a review:^27^. In other words, one might ask how much information about an individual’s PA skills is necessary in order to tailor a personalized reading curriculum. Individual differences in PA are predictive of reading development^15,20,28^, and training PA early on is useful for establishing a solid foundation for learning to read (e.g.,^29–31^, but it is also clear that PA is only one of a constellation of skills that contribute to ongoing differences in reading development^32^. For example, even though PA is a useful target for early intervention (e.g.,^33,34^, it is apparent that PA is not the only important dimension of variability. How PA should be considered in multifactorial models of reading development is still an active area of research^5,11,35–41^.

One of the difficulties in resolving the constellation of factors that interact to confer risk for reading difficulties such as dyslexia is that many findings represent small samples, in unique lab situations, and therefore are not necessarily generalizable, replicable, or representative of the general population^42,43^. A major barrier to scale research is personnel requirements, like the need for trained test administrators. This problem is exacerbated for multifactorial models where a researcher wants to collect measures of PA alongside a battery of other measures in a large and diverse sample. The most well-known and standardized PA assessments (e.g., PAT-2:NU^44^, CTOPP-2^45^, PPA^46^, require a test administrator to read instructions and interpret responses. Administering these PA assessments requires a high level of expertise, and participant responses are often difficult and subjective to score. Moreover, development of the PA skills that are measured by these verbal (i.e., expressive) tasks often goes hand in hand with development of articulatory skills, and confidence in expressive language more generally, leading to additional sources of variability in scoring^47^. Finally, PA tests are often long and tedious, and different subtests have variable instructions. This necessitates multiple training items for each subtest, making testing complicated and straining for the limited attention skills of young children.

Online experiments have grown in popularity as an alternative to one-on-one testing because they: (a) increase the efficiency of collecting larger, more diverse and representative samples, (b) reduce experimenter effects/biases, and (c) can be short, gamified, and engaging for young children^48–51^). Although implementations of online tests are still limited in developmental and educational research, first steps are being taken in reading and PA research. A first example is EarlyBird Education^52^. This self-administered, tablet-based game generates a literacy profile for early elementary school students. EarlyBird is now used as a screener for dyslexia (and reading difficulties more broadly). A second example is the Access to Literacy Assessment System–Phonological Awareness (ATLAS-PA^53^, a computer adaptive receptive measure of PA designed for children with speech and/or language impairments that is administered using an online platform. This work confronts the limitations of expressive PA tasks, for children who might not have impaired PA skills, yet are not able to show them through verbal tasks (i.e., children with speech disorders), and validates the use of a receptive PA task, showing its value for both children with and without speech and/or language impairments. Finally, our previous work introduced the Rapid Online Assessment of Reading Ability (ROAR, https://roar.stanford.edu,^51^). This is a self-administered, lightly gamified assessment of single word recognition (SWR) that overcomes the constraints of resource intensive, in-person reading assessment, and provides an efficient and automated tool for online research into the mechanisms of reading (dis)ability. ROAR-SWR is a short (~ 5 min), age-appropriate, lexical decision task that correlates highly with in-person standardized measures of reading ability like the Woodcock-Johnson Letter-Word Identification test (r = 0.91^54^. The success of this accurate, reliable (Cronbach’s α = 0.98), expedient and automated online measure of single word reading ability led to the present work on the development of the Rapid Online Assessment of Reading—Phonological Awareness (ROAR-PA).

Our goals were to (1) develop an efficient, online task that measures PA skills without verbal responses, (2) validate this measure against widely used in-person PA tests like the Comprehensive Test of Phonological Processing-2 (CTOPP-2^45^; and (3) assess the utility of this measure in a classroom setting. In contrast to other measures of PA, ROAR-PA was designed as an engaging game for young children such that it would not require a test administrator. Items are narrated by animated characters, and the participant selects responses with a touch screen (or mouse), meaning that scoring is completely automated. The intention of implementing a PA assessment to run in the web-browser was to measure phonological processing skills efficiently and accurately across a broad age range—kindergarten through 8th grade. Specifically, we were interested to see (a) whether a receptive (prerecorded 1-interval 3-alternative-forced-choice,1I-3AFC) online task could accurately capture comparable PA skills to the CTOPP-2 which requires verbal responses, (b) whether different subtests measure unique latent traits of PA, (c) whether the test is developmentally appropriate from kindergarten up to 8th grade and (d) whether it is appropriate for large scale assessment in schools and predictive of future reading skills. In Part 1, we assess the feasibility of using an online PA measure for five different subtests: First Sound Matching (FSM), Last Sound Matching (LSM), Rhyming (RHY), Blending (BLE) and Deletion (DEL) (See Methods section for more details). In Part 2, Factor Analysis is used to examine the coherence of the subtests in measuring a latent PA construct. Based on item response theory (IRT) we then select suitable items spanning different difficulty levels for an efficient assessment. In Part 3 we look at the predictive value of our task for reading performance and develop an automated score report. And, finally, in Part 4 we test the predictive validity of these innovations by implementing ROAR-PA in 50 first and second-grade classrooms and examining sensitivity and specificity of this measure for risk classifications based on individually administered reading assessments.

## Results

### Part 1: feasibility of online PA measure, subtest and age selection

#### Validation of ROAR-PA composite score

**Figure 1:**
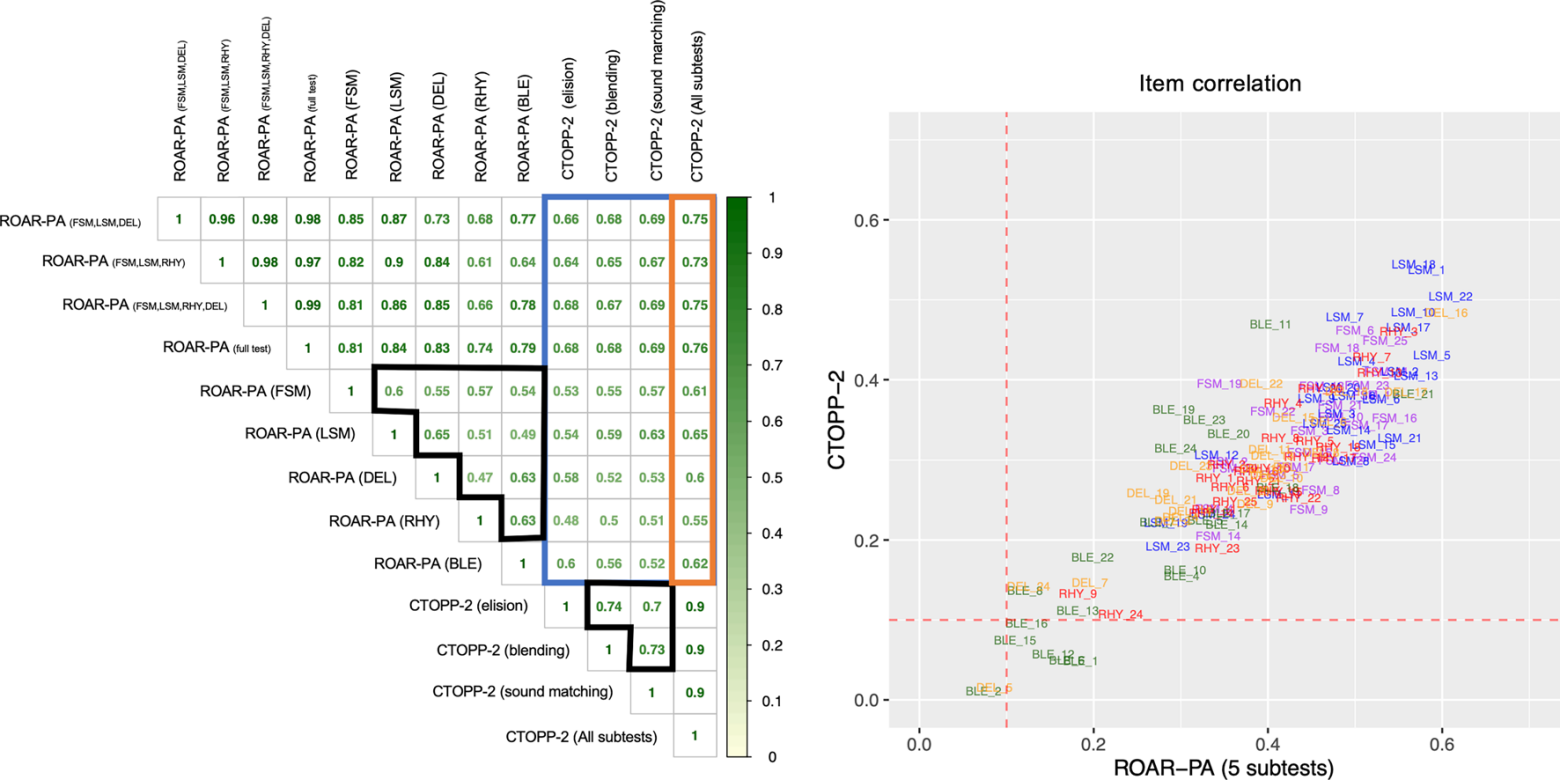
Left: A Pearson correlation matrix between the 5 ROAR-PA subtests (% correct), CTOPP-2 subtests (raw scores), and overall (raw) scores on both tasks, of the original cohort that completed all 5 subtests (N = 143). Orange box: correlation coefficients between ROAR-PA and overall CTOPP-2, Blue Box: correlation coefficients between ROAR-PA and subtests of CTOPP-2, Black boxes: correlation boxes within subtests of ROAR-PA and subtests of CTOPP-2. Right: Item Correlation Analysis: For each stimulus, we plot the point-biserial correlation between performance on the item and ROAR-PA accuracy (x-axis) as well as the correlation between performance on the item and CTOPP-2 raw score (y-axis). Items with low correlations (threshold r ≤ .10; dotted red line) with overall test performance or CTOPP-2 performance were removed from the test.

To validate the feasibility of a web-browser based PA task (containing 5 subtests: FSM, LSM, RHY, BLE, and DEL) that only requires clicks/touchscreen responses, we tested 143 participants (Age: 3.87–13.00, μ = 7.13, σ = 1.89; Sex: 67 F, 76 M) and performed a correlation analysis between each ROAR-PA subtest and the well-established standardized CTOPP-2. The results (Fig. 1, left panel) revealed strong correlations between the CTOPP-2 and all ROAR-PA subtests: LSM (r = 0.65), DEL (r = 0.62), FSM (r = 0.61), RHY (r = 0.60), and BLE (r = 0.55). The correlations between the subtests (0.47 $\le$ r $\le$0.65) of the ROAR-PA composite were slightly lower and more variable than we would have expected based on the correlation of the CTOPP-2 subtests in this dataset (0.70 $\le$ r $\le$ 0.73). This could indicate that ROAR-PA subtests tap into different latent constructs, or it could reflect the reliability of each subtest. Each subtest, except for BLE, showed high internal consistency based on Cronbach’s α (LSM: α = 0.92, CI_95_ = [0.89; 0.93], FSM: α = 0.90, CI_95_ = [0.87; 0.93], RHY: α = 0.86, CI_95_ = [0.81; 0.89], DEL: α = 0.84, CI_95_ = [0.77; 0.88], BLE: α = 0.70, CI_95_ = [0.57; 0.78]) and the composite scores of both CTOPP-2 (α = 0.88, CI_95_ = [0.85 ; 0.91]) and ROAR-PA (α = 0.85, CI_95_ = [0.80; 0.89]) had good (0.8 ≤ α < 0.9) internal consistency.

To optimize ROAR-PA as a valid screening tool we sought to create a composite score that best approximated the CTOPP-2 composite index. To do so, we created a linear model with the CTOPP-2 scores as the dependent variable and the scores of each individual subtest as predictor variables. This model (CTOPP-2 ~ FSM + LSM + RHY + BLE + DEL) showed that the subtests FSM ($\beta$ = 0.79; t = 2.33; *p* = 0.02), LSM ($\beta$ = 1.07; t = 3.92; *p* < 0.001), and DEL ($\beta$ = 1.27; t = 3.13; p = 0.002) were significant predictors of the CTOPP-2 scores, but the subtests RHY ($\beta$ = 0.44; t = 1.13; p > 0.10), and BLE ($\beta$ = 0.55; t = 0.90; p > 0.10, were not. We then used a Likelihood Ratio test to determine the influence of these non-significant subtests in our CTOPP-2 prediction, by comparing the full model, as described above, to a model without BLE (4 ROAR-PA subtests: FSM, LSM, RHY, DEL); and a model with only the 3 significant subtests (FSM, LSM, DEL). We found no significant differences in model predictions between the full model and the 4 subtest model ($\chi$^2^ = 0.85; p > 0.10), nor the full model and the 3 subtest model ($\chi$^2^ = 2.05; p > 0.10), suggesting that the three subtests (FSM, LSM, DEL) are sufficient to obtain an accurate PA composite that approximates the CTOPP (R^2^ = 0.57).

These findings are corroborated by interpreting the Pearson correlation coefficients between ROAR-PA and CTOPP-2. Although the highest correlation was reported by summing the scores on all 5 ROAR-PA subtests (r = 0.76)., a composite score based on 4 (FSM, LSM, DEL,RHY) or 3 ROAR-PA subtests (FSM, LSM, DEL) was equally correlated with CTOPP-2 (r = 0.75). The 3-subtest composite and 4-subtest composite both achieved good reliability as well: Cronbach’s alpha of α_4_subtests_ = 0.84, CI_95_ = [0.77; 0.88] and α_3_subtests_ = 0.78, CI_95_ = [0.67; 0.84] respectively. As convergent validity greater than r = 0.70 is recommended to reflect whether two measures capture a common construct^55^, it can be concluded that all possible composite scores (5, 4, and even 3 subtests) suffice to capture PA skills.

Furthermore, an item analysis, based on the correlations between the item responses of ROAR-PA for each of the 123 test items and CTOPP-2 scores, showed that performance on items from the subtest LSM were especially highly correlated with overall ROAR-PA performance and CTOPP-2 performance (Fig. 1, right panel). This suggests LSM items are most informative about overall PA abilities. Items from the BLE subtest were least informative: the correlation between most blending items and ROAR-PA total score and CTOPP-2 total score was close to zero.

#### Age selection

**Figure 2:**
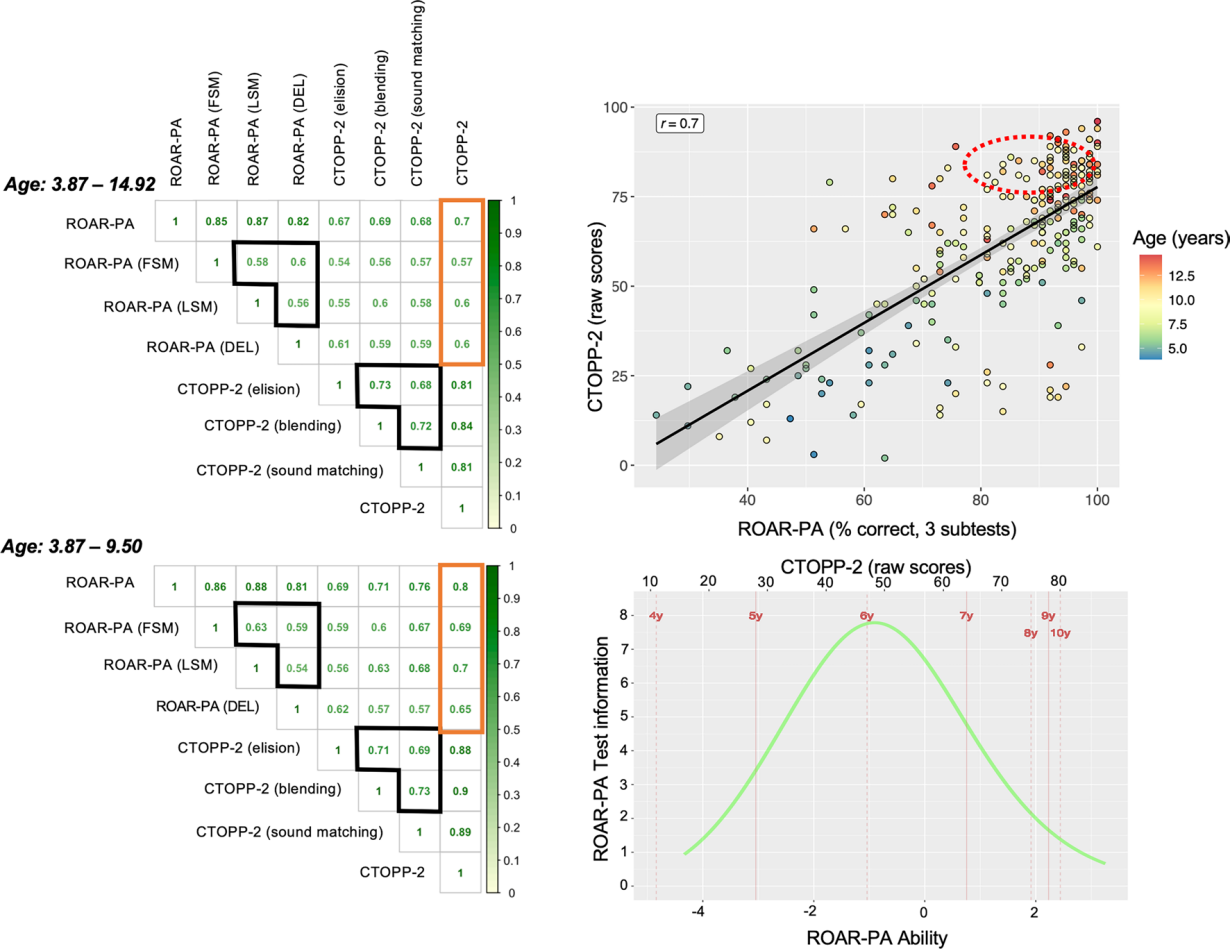
Left: Pearson correlation matrix between the 3 selected ROAR-PA subtests (% correct) and between the CTOPP-2 subtests (raw scores) and overall scores on both tasks for all children (age 3.87–14.92 years; N = 266—top) and for a subset of children, based on the limited age-selection (age 3.87–9.50 years; N = 145—bottom). Orange box: correlation coefficients between ROAR-PA and overall CTOPP-2, Black boxes: correlation boxes within subtests of ROAR-PA and subtests of CTOPP-2. Right top: Pearson correlation plot between the ROAR-PA (% correct) and between the CTOPP-2 (raw scores), for all children (N = 266) age 3.87–14.92 years. The red dotted oval points to the ceiling effect of the oldest children. Right bottom: Test information functions for the ROAR-PA. The x-axis shows ability estimates based on the Rasch model. The upper x-axis shows the estimated CTOPP-2 raw score equivalent based on the linear relationship between ability estimates and CTOPP-2 scores. The pink lines indicated age equivalents for CTOPP-2 scores (based on the CTOPP-2 manual). Test information is high for participants scoring between 4.5 and 9.5 years age equivalent on the CTOPP-2.

After selecting 3 subtests that make an efficient and reliable ROAR-PA composite score, we collected ROAR-PA data for an additional group of 127 participants (including mostly older children) resulting in a total of 270 participants (Age: 3.87–14.92, μ = 9.12, σ = 2.71; Sex: 125 F, 145 M) who completed ROAR-PA FSM, LSM, and DEL subtests. Of these participants, 266 were also administered the CTOPP-2 PA assessment. The Pearson correlation analysis with the CTOPP-2 for this extended group of participants resulted in an overall correlation between CTOPP-2 and ROAR-PA composite (3 subtests) of r = 0.70 (as opposed to r = 0.75 in the initial sample of participants). The correlation between the CTOPP-2 and the individual subtests also went down for FSM and LSM (Fig. 2. Left top). The decrease in correlation likely reflected ceiling effects in older participants (Fig. 2, right top).

To examine the effect of age on the correlation between ROAR-PA and CTOPP-2, we split our sample into 3 different age bins (3.87–6.99 years old (N = 71), 7.00–9.99 years old (N = 91), 10.00–14.92 years old (N = 104)). We found a correlation coefficient between the composite scores of ROAR-PA and CTOPP-2 of r = 0.79 (CI_95_ = [0.68; 0.86], Cronbach’s α = 0.88) for the youngest group, r = 0.69 (CI_95_ = [0.56; 0.78], Cronbach’s α = 0.79) for the middle group, and r = 0.31 (CI_95_ = [0.13; 0.48], Cronbach’s α = 0.65) for the oldest group of children. Further analysis (correlation and Rasch analysis) provided an ideal age range up to 9.50 years old for the ROAR-PA (Fig. 2, bottom left and right), leading to a Pearson correlation coefficient of r = 0.80 (CI_95_ = [0.73; 0.85], Cronbach’s α of 0.80) between the ROAR-PA composite and CTOPP-2, and an increase of the correlations for individual subtests (FSM, LSM, DEL) to the CTOPP-2. This indicates that the ROAR-PA in its current form is predictive of PA skills for children in (pre-) kindergarten through fourth grade (Fig. 2., bottom right) but has ceiling effects above fourth grade. Interestingly, the correlation analysis in our sample shows a similar effect for the CTOPP-2 scores, indicating both PA tasks (ROAR-PA and CTOPP-2) are most suited for younger children.

### Part 2: factor, item, and difficulty analysis

#### Factor analysis (FA)

To evaluate the dimensionality of the ROAR-PA assessment we used exploratory FA with oblique rotation. FA poses the question of whether there is evidence that all of these items are measuring the same underlying phonological processing ability, or whether the items of these subtests better represent separable (but correlated) dimensions of PA.

**Figure 3:**
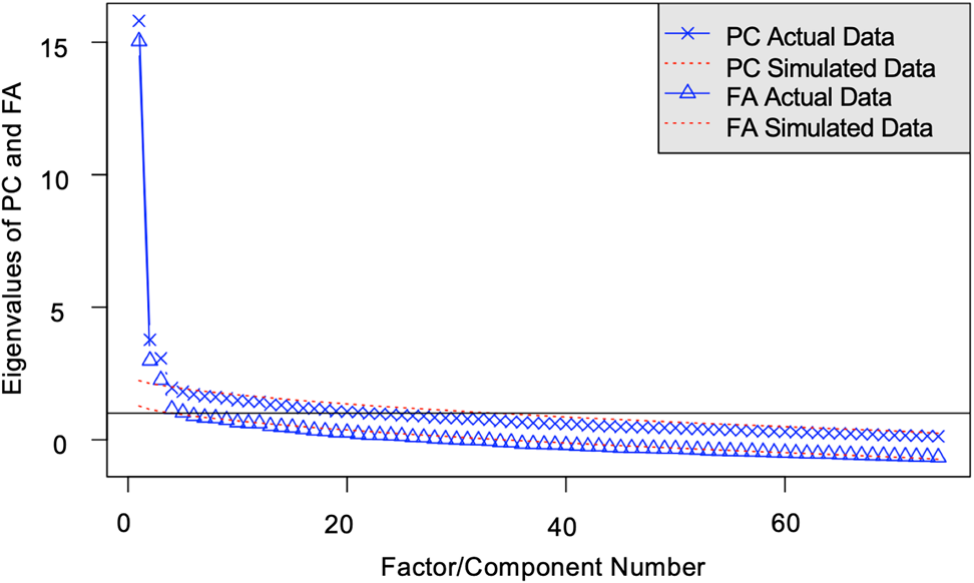
Parallel Analysis of Scree plots of 74 items on three subtests (FSM, LSM, DEL). Inspection of the scree plot suggests three factors before the amount of variation represented by the eigenvalues flattens out. The dotted red lines represent extracted eigenvalues from data sets that are randomly created; there are three factors with observed eigenvalues that are larger than those extracted from the simulated data.

Our results suggest a multi-dimensional framework. First, the scree plot (Fig. 3) of the different items (N = 74) on these three subtests (FSM, LSM, DEL) indicate three factors before the rate of decrease flattens. Second, the magnitude of the loadings for the three-factor model are larger than the one-factor model. Finally, examining the factor loadings (Table 1), the items from each of the three subtests cleanly separate into separate factors (with the exception of a single item: FSM_13).

**Table 1:** Exploratory Factor Analysis with all subtest items (N = 74) loaded on 1,2 or 3 factors.

Items	One factor	Two factors		Three factors		
	Factor 1	Factor 1	Factor 2	Factor 1	Factor 2	Factor 3
FSM_1	0.49	0.52		0.49		
FSM_2	0.43			0.36		
FSM_3	0.48	0.49		0.3		
FSM_4	0.38	0.6		0.5		
FSM_5	0.5	0.38		0.32		
FSM_6	0.47	0.49		0.45		
FSM_7	0.48	0.51		0.53		
FSM_8	0.44	0.35		0.47		
FSM_9	0.41	0.37		0.54		
FSM_10	0.5	0.49		0.54		
FSM_11	0.56	0.64		0.58		
FSM_12	0.5	0.57		0.31		0.33
FSM_13	0.47	0.65				0.61
FSM_14	0.38	0.4		0.57		
FSM_15	0.5	0.47		0.52		
FSM_16	0.53	0.33		0.51		
FSM_17	0.5	0.62		0.33		0.37
FSM_18	0.56	0.57		0.62		
FSM_19	0.38	0.33		0.63		
FSM_20	0.36					
FSM_21	0.52			0.72		
FSM_22	0.48	0.52		0.53		
FSM_23	0.46	0.48		0.52		
FSM_24	0.52	0.5		0.62		
FSM_25	0.59	0.6		0.62		
LSM_1	0.55		0.53		0.52	
LSM_2	0.55		0.54		0.51	
LSM_3	0.39		0.35		0.33	
LSM_4	0.48		0.6		0.57	
LSM_5	0.53		0.63		0.61	
LSM_6	0.52		0.58		0.54	
LSM_7	0.51		0.55		0.52	
LSM_8	0.48		0.52		0.5	
LSM_9	0.48		0.35		0.34	
LSM_10	0.56		0.49		0.46	
LSM_11	0.42		0.45		0.43	
LSM_12	0.31	0.32				
LSM_13	0.55		0.64		0.62	
LSM_14	0.46		0.63		0.6	
LSM_15	0.53		0.58		0.55	
LSM_16	0.45		0.66		0.63	
LSM_17	0.54		0.63		0.61	
LSM_18	0.52		0.55		0.53	
LSM_19			0.51		0.52	
LSM_20	0.48		0.52		0.5	
LSM_21	0.49		0.48		0.46	
LSM_22	0.59		0.65		0.64	
LSM_23			0.42		0.41	
LSM_24	0.4		0.63		0.64	
LSM_25	0.4		0.5		0.5	
DEL_1	0.39	0.62				0.7
DEL_2	0.3	0.33				0.36
DEL_3	0.33	0.32				0.42
DEL_4	0.31	0.42				0.55
DEL_5						0.47
DEL_6	0.39	0.52				0.5
DEL_7						0.39
DEL_8	0.5	0.68				0.54
DEL_9	0.35	0.38				0.55
DEL_10	0.43	0.31				0.33
DEL_11	0.47	0.41				0.34
DEL_12	0.36					
DEL_13	0.36					0.34
DEL_14	0.37	0.43				0.51
DEL_15	0.41	0.36				
DEL_16	0.62	0.48				0.37
DEL_17	0.55	0.32				0.43
DEL_18	0.44					0.33
DEL_19	0.35	0.35				
DEL_20	0.34					
DEL_21	0.34	0.44				0.47
DEL_22	0.43	0.47				0.47
DEL_23	0.33					
DEL_24						

An oblique rotation was used as we are not assuming orthogonal relationships between factors, and loadings that are less than .30 are suppressed for the ease of interpretation. The table supports the three-factor model and loadings are overall larger compared to the one-factor model.

#### Item analysis

**Figure 4:**
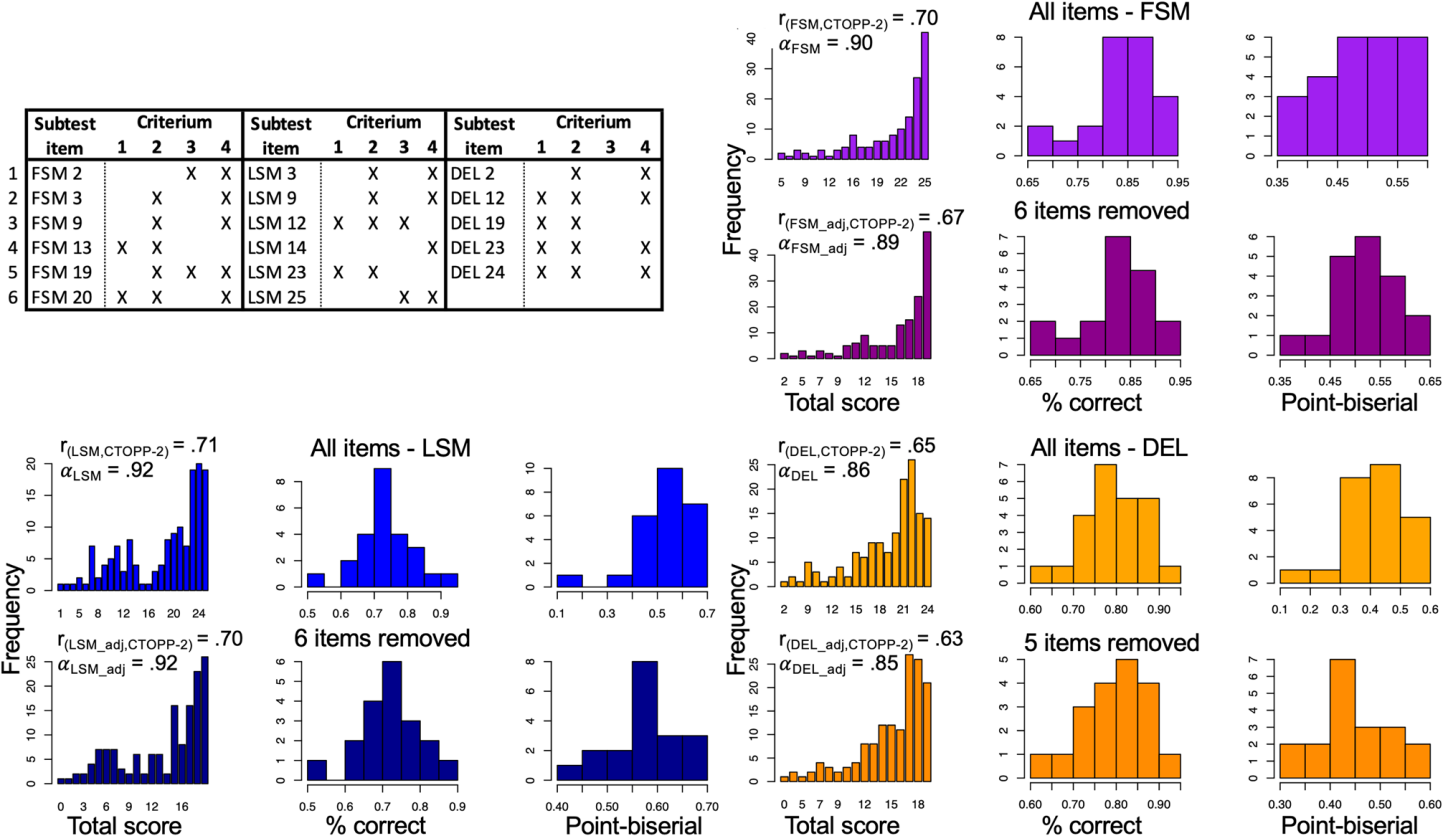
Item analysis: Rasch models with .333 fixed guess rate, including students between 3.87 and 9.50 years old, that were not excluded based on clicking or foil patterns for the three remaining subtests (FSM, LSM and DEL). Left Top: Deleted items based on a minimum of meeting 2/4 criteria. Right top & Bottom: Representation of correlation with CTOPP-2, Cronbach's α, Distributions of % correct values and the point-biserial correlations for all items per subtest and for the subtests with items deleted based on the four criteria.

In a second step we identified a subset of items from ROAR-PA to remove in order to both improve model fit as well as reduce the length of the assessment. Given the evidence for a multi-dimensional framework, we proceeded by calibrating a Rasch Model separately for each of the subtests (FSM, LSM and DEL). In this IRT analysis we included data for all participants between 3.87 and 9.50 years old. For each subtest we reviewed four criteria, compiled from both the factor analysis and Rasch Model item fit statistics, to determine the best subset of items: (1) Does the item load on the subtest factor with a relationship > 0.30?^56^ (2) Does the item resemble a functional form when looking at empirical plots?^57^ (3) Is the item flagged based on Rasch model fit statistics?^58^ (4) Finally, as we want items to be informative and not redundant, is the item located near two or more items based on difficulty distribution, to create a test length that seems appropriate for children's attention spans (Fig. 4, left top)?

Analysis of the FSM subtest (Fig. 4., right top) suggested removing 6 of the 25 items. After removing these items, no major degradation or change in the key item statistics for this assessment was observed. Cronbach’s α remained high (α_FSM_all_items_ = 0.90, CI_95_ = [0.87; 0.93] & α_FSM_adjusted_ = 0.89, CI_95_ = [0.85; 0.92]), and the distributions of the proportion-correct values and the point-biserial correlations for all items remained similar. The correlation (Fig. 2, left bottom) between FSM total scores and CTOPP-2 stayed about the same (r_FSM_all_items_ = 0.69, r_FSM_adjusted_ = 0.67). Analysis of the LSM subtest (Fig. 4., left bottom) also suggested removing 6 out of 25 items. Similar to FSM, Cronbach’s α of LSM remained high (α_LSM_all_itemsl_ = 0.92, CI_95_ = [0.90; 0.94] & α_LSM_adjusted_ = 0.92, CI_95_ = [0.90; 0.93]), the distributions of the proportion-correct values, the point-biserial correlations, and the correlation between the total scores and the CTOPP-2 remained similar (r_LSM_all_itemsl_ = 0.70, r_LSM_adjusted_ = 0.70). Analysis of the DEL subtest (Fig. 4., right bottom) indicates removal of 5/24 items. Again, Cronbach’s α remained high (α_DEL_all_items_ = 0.86, CI_95_ = [0.79; 0.89] & α_DEL_adjusted_ = 0.85, CI_95_ = [0.78; 0.89]), the distributions of the proportion-correct values, the point-biserial correlations, and the correlation between the total scores and the CTOPP-2 remained similar (r _DEL_all_items_ = 0.65, r_DEL_adjusted_ = 0.63).

This Rasch item analysis suggests that every subtest of this ROAR-PA task has a good (DEL) to excellent (FSM, LSM) internal consistency, based on Cronbach’s α, with a strong correlation of every subtest (r > 0.65) to the overall CTOPP-2 scores. Item analysis based on meeting at least 2/4 suggested criteria, results in 19 items per subtest, and an overall task of 57 items + 2 practice items per subtest.

#### Difficulty analysis

**Figure 5:**
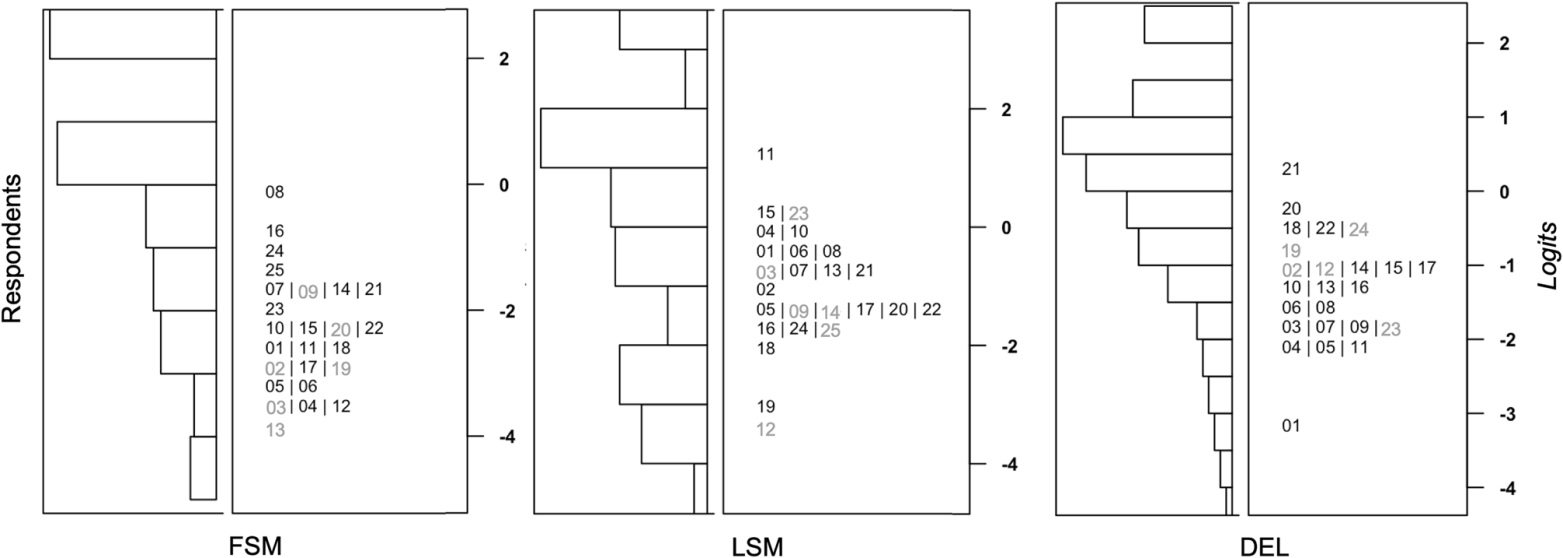
Difficulty analysis: Item-person maps (or “Wright Map”) for the three subtests. Per subtest, the distribution of the ability of the students is plotted on the left-hand side (higher ability is closer to the top of the map). The distribution of the item difficulty is plotted on the right-hand side. The deleted items from the item analysis are grayed out.

ROAR-PA items were designed to span different theoretical levels of difficulty (e.g.,^9^^,^^17^. For the DEL subtest, difficulty levels were based on manipulation of (1) words and syllables (item 1–8), (2) onset and rimes (item 9–16), or (3) phonemes in the middle of the word (item 17–24). For FSM and LSM we could not follow these levels, as the task itself focuses on the first or last phoneme(s) of the word. We tried to create difficulty levels by manipulating single phonemes (level 1: item 1–16) or a single phoneme in a phoneme cluster (level 2: item 17–25). Surprisingly, based on the Rasch Model item-person maps for the three subtests (Fig. 5), we only found that the subtest DEL approximately follows the expected difficulty pattern. This analysis also showed that for FSM most items are closer to the lower-range of ability, for LSM and DEL most items are close to the mid-range of ability.

#### Summary

Analysis Part 1 and Part 2 led to the development of a new version of the ROAR-PA task (ROAR-PA_v.2), designed for children kindergarten to 5th grade. The task is a receptive PA assessment (1I-3AFC) consisting of 57 items (19 per subtest), divided into three subtests: First Sound Matching (FSM), Last Sound Matching (LSM) and Deletion (DEL). Each of these subtests load on their own factor, and together they span a range of abilities of phonological processing. The ROAR-PA_v.2 presents 2 training items per subtest, followed by the test items in a predetermined difficulty order (based on the IRT analysis). Selection of the most appropriate age group (kindergarten to 5th grade) and the most reliable items (19 per subtest) results in a highly reliable (Cronbach’s α for ROAR-PA_v.2 composite = 0.96; CI_95_ = [0.95; 0.97]) automated self-scored task that takes, on average, 12 min.

### Part 3: correlation to reading skills, and score reports

The ultimate goal of developing an efficient, online PA task is to create a task that (a) predicts reading performance and (b) provides actionable information about the individual’s PA skills.

To accomplish the first goal, we correlate ROAR-PA performance to performance on another task of the ROAR test battery: ROAR-Single Word Recognition (SWR)^51^ as well as reading skills assessed with two of the most widely used standardized, individually administered measures of reading, the Woodcock-Johnson Letter-Word-Identification (WJ LW-ID) and Word-attack (WJ-WA) tests^59^. Reading scores can only be assessed after children learn how to read, therefore we only acquired reading performance of 111 participants (Age: 5.66–9.50, μ = 7.67, σ = 1.13,Sex: 59 F, 52 M) on the WJ and ROAR-SWR. Results show a Pearson correlation coefficient of r = 0.47 between WJ LW-ID and the composite score of the ROAR-PA task, and for the individual subtests of ROAR-PA: r_FSM_ = 0.32, r_LSM_ = 0.33, r_DEL_ = 0.50. Between WJ-WA and the ROAR-PA composite score a Pearson correlation coefficient of r = 0.50 was reported (r_FSM_ = 0.39, r_LSM_ = 0.34, and r_DEL_ = 0.53), and r = 0.53 between ROAR-SWR and the ROAR-PA task (r_FSM_ = 0.41, r_LSM_ = 0.40, and r_DEL_ = 0.47). For sake of comparison, CTOPP-2 was correlated at r = 0.66 with WJ-WA, r = 0.73 with WJ LW-ID, and r = 0.55 with ROAR-SWR.

**Figure 6:**
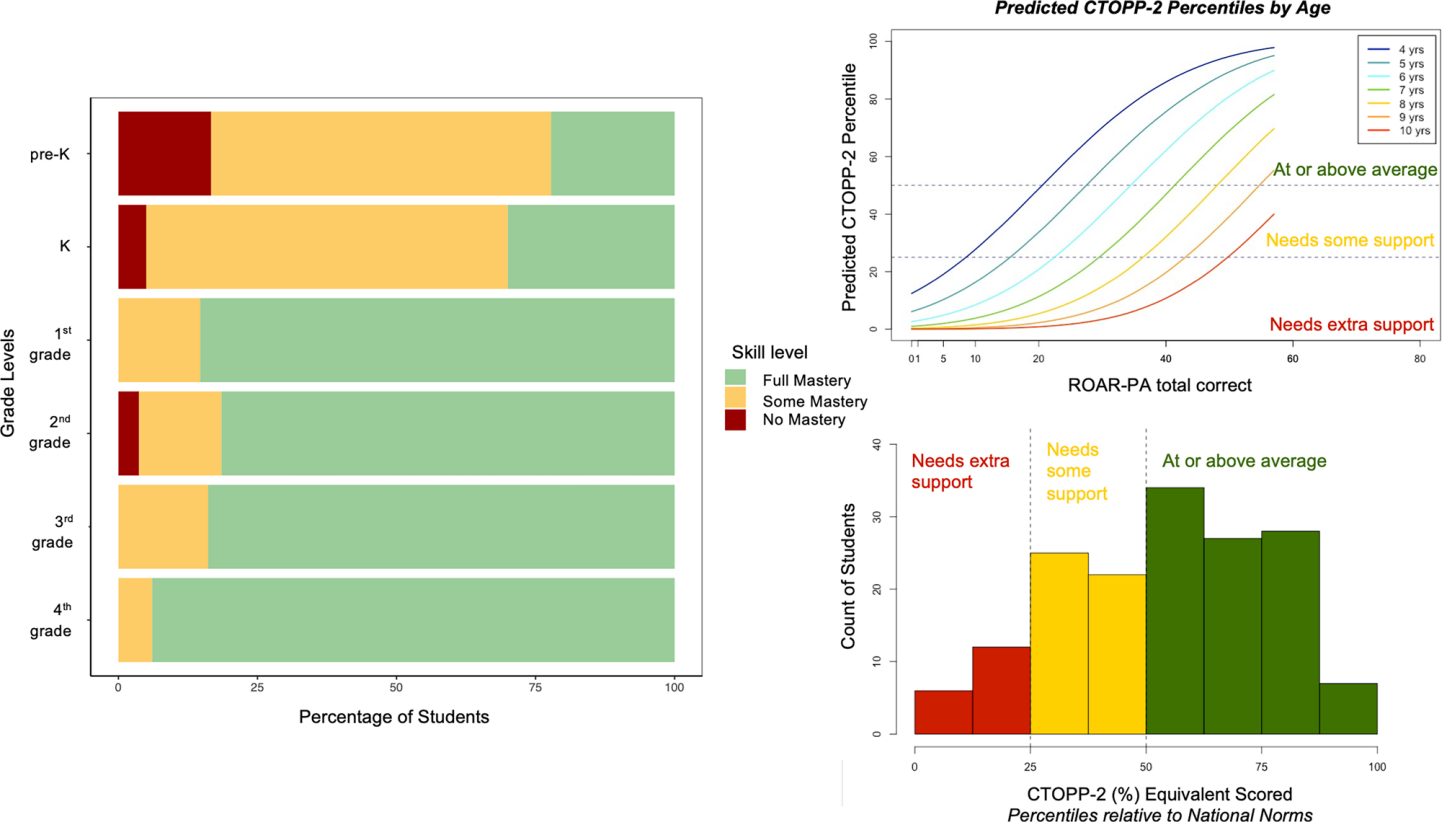
Left: Different mastery levels represented as percentage of students by grade. Results show (as expected) that mastery increases by grade. Right top: Predicted CTOPP-2 percentiles for the ROAR-PA scores by age. Red line shows ceiling effects of the test for 10-year-olds, indicating that the ability range is uninformative for the oldest participants. Right bottom: Distribution of the estimated CTOPP-2 percentile scores, based on ROAR-PA performance, identifying children at risk (< pc 25), some risk (pc 25–50) or doing well (> pc 50).

To accomplish the second goal, we used a diagnostic classification model (DCM) to develop a ROAR-PA score report that provides information about mastery of each PA subskill. More specifically, we use a specific DCM known as the deterministic-input, noisy-and-gate model, or the DINA model^60^. The score report presents information about N = 163 (Age: 53.87–9.92, μ = 7.30, σ = 1.63,Sex: 80 F, 83 M), from pre-k to the end of fourth grade. This analysis used the 57 final items selected in Part 2. Mastery of a subtest was defined as scoring > 50% correct per subtest. The average ROAR-PA score was 44.76 (out of 57) (min = 12, max = 57, σ = 11.06). Participants’ mastery of the three skills (FSM, LSM and DEL) was quantified into three categories: Full Mastery (i.e., mastery of all 3 subtests), Some Mastery (i.e., mastery of 1 or 2 subtests), or No Mastery (i.e., mastery of 0 subtests). The results of these analyses show that with increasing grade level, the level of mastery improves. There is substantial variability in mastery at each grade level indicating that ROAR-PA provides additional information for identifying students who may benefit from additional support in specific PA skills (Fig. 6., left).

Furthermore, students’ performance can be interpreted in relation to percentile scores representing each student’s phonological awareness skills relative to CTOPP-2 national norms, (Fig. 6., right), by fitting a regression model relating ROAR-PA scores to CTOPP-2 scores. The distribution of the estimated CTOPP-2 percentile scores allows categorization of the students as “needs extra support” (children performing below the 25th percentile; N = 18), “needs some support” (pc 25–50; N = 47), and “at or above average” (> pc 50; N = 96), indicating that all children $\le$ pc 50, should be monitored or followed-up with other measures.

### Part 4: assessment of ROAR-PA_v.2 in school districts

After developing an efficient PA screener that can categorize performance by a potential need for support, ROAR-PA should be evaluated on a larger scale, outside of a “research settings”. To assess the value of ROAR-PA as a screener in a classroom setting we ran a study in collaboration with a collection of California schools to a) investigate feasibility of district-wide administration and b) assess predictive validity of the automated ROAR measures against standard-of-practice individually administered reading assessments. The new version of the ROAR-PA task (ROAR-PA_v.2: 3 subtests, 57 items, and early stopping criteria—see Methods), was used across six schools in the state of California (N = 901; Grade 1–5). Students completed ROAR-PA and ROAR-SWR on chromebooks in their classroom and all students in a class completed the assessments simultaneously. Additionally, students in 1st (N = 345) 2nd (N = 237) and grade were individually administered the Fountas and Pinnell Benchmark Assessment (F&P^61^; by their classroom teacher as part of standard practice. Even though the validity of F&P reading levels has been questioned by researchers for a variety of reasons^62,63^, F&P classifies children’s reading scores in categorical reading levels (i.e., AA−Z +), to then use them as guided reading levels in book choices for children, and therefore remains one of the most widely used assessment systems in educational practice. Thus, analyzing the correspondence between ROAR and F&P has utility for schools that currently rely on F&P even though additional validation of ROAR against accepted research measures is still warranted.

**Figure 7:**
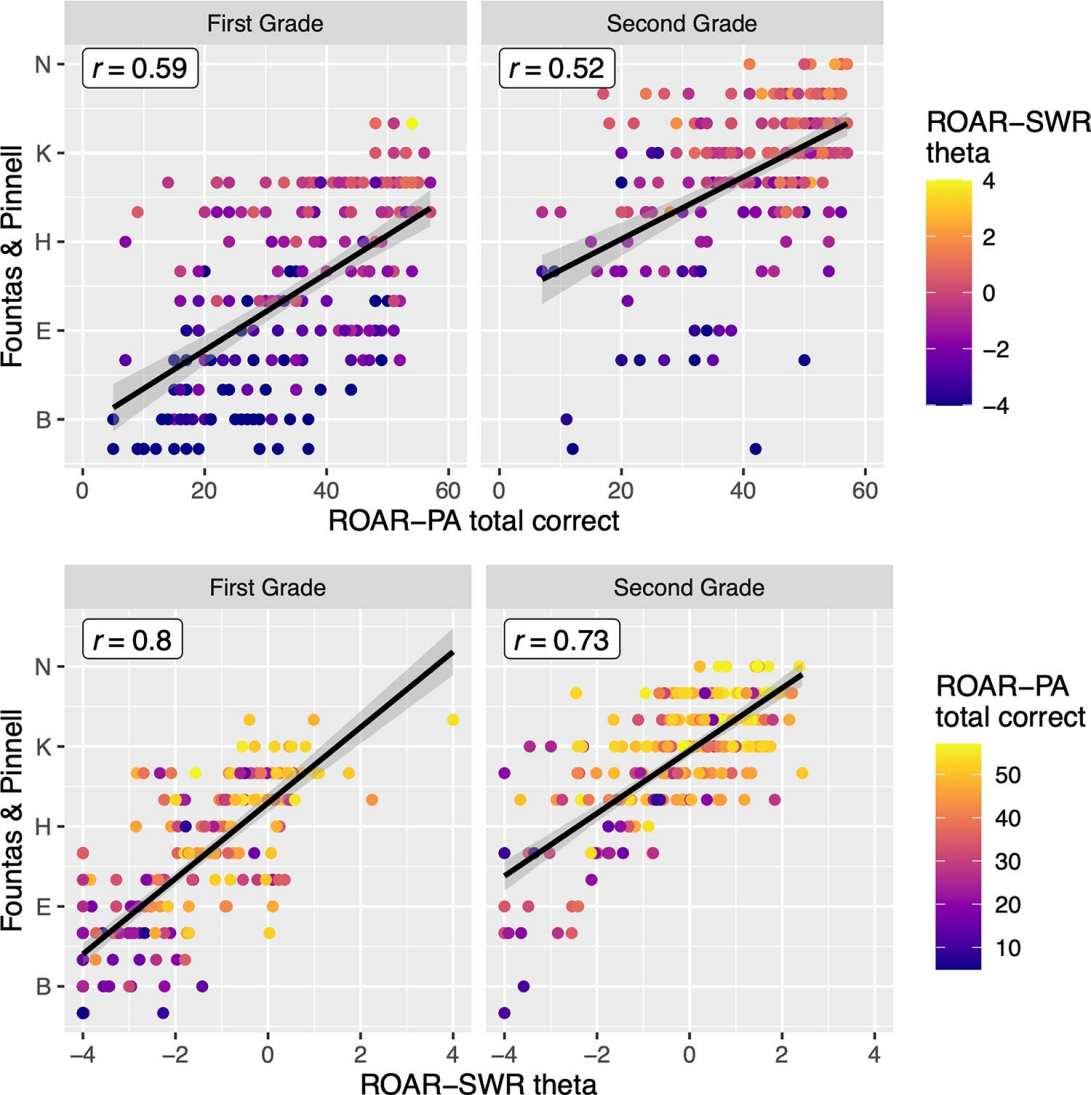
Top: Pearson correlation plot between total scores of the ROAR-PA and Fountas & Pinnell Benchmark scores in 1st and 2nd grade. The colorbar shows ROAR-SWR theta score for each point. Bottom: Pearson correlation plot between the ROAR-SWR theta scores and Fountas & Pinnell Benchmark scores in 1st and 2nd grade. The colorbar shows ROAR-PA composition score for each point. The colormap of these plots indicates that high ROAR-PA scores correspond to high ROAR-SWR scores, but ROAR-PA also provides additional information beyond ROAR-SWR.

First, ROAR-PA showed a moderate correlation with F&P scores in 1st grade (Fig. 7; r = 0.59) and 2nd grade (r = 0.52). This is in line with previously established correlations linking PA to other measures of reading ability in early elementary school (r = 0.46^12^, r = 0.41 (real words)—r = 0.43 (non-words)^64^.

Second, we ask whether a linear regression model with ROAR-PA composite scores provides additional information about F&P reading level above and beyond ROAR-SWR scores. A regression model with ROAR-PA and grade level predicted 56% of the variance in F&P; ROAR-SWR and grade level predicted 74% of the variance in F&P; ROAR-PA, ROAR-SWR and grade level predicted 76% of the variance in F&P. A model comparison indicated that the model with ROAR-PA, ROAR-SWR and grade was a significantly better fit than the model with just ROAR-SWR and grade (F_(1,448)_ = 46.74, p < 0.001). Thus, ROAR-PA and ROAR-SWR provide complementary information.

Third, we examined classification accuracy of ROAR-PA and ROAR-SWR for students who were deemed “at risk” versus “not at risk” by their school district based on F&P scores. First grade students were deemed at risk if they scored below E and 2nd grade students were deemed at risk if they scored below J. In 1st grade, a generalized additive model (GAM^65^; with ROAR-PA achieved an area under the curve (AUC) = 0.82, ROAR-SWR achieved an AUC = 0.91, and a GAM with ROAR-PA and ROAR-SWR achieved an AUC = 0.93. In 2nd grade, ROAR-PA achieved an AUC = 0.79, ROAR-SWR achieved an AUC = 0.87 and a model with ROAR-PA and ROAR-SWR achieved an AUC = 0.91. Thus the ROAR assessments are able to accurately classify most students deemed at risk based on F&P Benchmark scores (Table 2) and a model combining ROAR-PA and ROAR-SWR scores performed better than either score on its own.

**Table 2:** Sensitivity and specificity of ROAR as a screener for reading difficulties.

Grade	Predictors	Accuracy	Sensitivity	Specificity	AUC
1	s(SWR)	0.85	0.89	0.77	0.91
1	s(PA)	0.76	0.86	0.56	0.82
1	te(SWR, PA)	0.87	0.91	0.79	0.93
2	s(SWR)	0.84	0.91	0.65	0.86
2	s(PA)	0.77	0.92	0.36	0.79
2	te(SWR, PA)	0.84	0.92	0.60	0.91

Results are shown separately for first and second grade. The mgcv package in R was used to fit GAMs with predictors as specified in the predictors column. Combining ROAR-PA with ROAR-SWR achieved exceptional sensitivity in both 1st and 2nd grade.

Finally, we examined the classification accuracy of ROAR-PA and ROAR-SWR administered in 1^st^ grade for classifying F&P levels 8 months later in the fall of 2nd grade (N = 130). A GAM with ROAR-PA achieved an AUC = 0.70, ROAR-SWR achieved and AUC = 0.83, and a GAM with ROAR-PA and ROAR-SWR achieved an AUC = 0.84. These results demonstrate the promise of ROAR-PA and ROAR-SWR as screening tools in the typical classroom setting.

## Discussion

Phonological Awareness is an important skill at the foundation of reading development^12,13,15^^,^ often introduced in preschool or kindergarten, which can serve as a predictor for later reading performance, and a target for early interventions^66^^,^^67^). The present work developed and validated an automated, online measure of PA for use in research and practice (https://roar.stanford.edu/pa). Most PA assessments are time-consuming, resource intensive and subjective to score. Our primary goal was to evaluate the suitability and efficiency of an online PA assessment to both increase accessibility for practitioners and reduce bottle-necks to large-scale research studies. We found that in just 12 min, ROAR-PA achieved reliability akin to in person assessment (α = 0.96), and had predictive validity when administered in the classroom setting by teachers with little training, making this a highly-efficient screening and assessment tool for both practitioners and researchers.

The initial version of ROAR-PA included 5 subtests, modeled after subtests of well-known, standardized, in-person PA assessments^44^^,^^45^^,^^46^). Results from Part 1 indicated that a composite measure based on 3 subtests (First Sound Matching,FSM, Last Sound Matching; LSM and Deletion; DEL) was sufficient to characterize a wide range of PA skills and reliably predict performance on the CTOPP-2 (Fig. 2., right bottom). The fourth subtest (i.e., Blending) was not sufficiently discriminative and the subtest Rhyming did not add additional information to the ROAR-PA composite above and beyond what was measured by the other 3 subtests. IRT analysis helped to narrow down the assessment to a brief version with a max of 57 (1I-3AFC) PA items. Based on an analysis of (a) the correspondence between ROAR-PA and individually administered CTOPP-2 assessments, and (b) the reliability of ROAR-PA composite scores, we determined that ROAR-PA is ideal for children between 4 and 9.5 years old. This version of ROAR-PA spans a continuum of PA abilities across the three subtests, and serves as a quick measure of PA well-suited for screening in pre-k through fourth grade. Even though our score report analysis (Part 3) showed ceiling effects for older children, it is still worth conducting future research to examine whether ROAR-PA can detect PA difficulties in older children with reading disabilities.

Most PA measures rely on verbal responses. An important goal of the present work was to ascertain whether a purely receptive test (touch screen responses) could achieve a similar measure of PA without verbal responses. We reported a strong correlation (r = 0.74) between the ROAR-PA and the well-established standardized “Comprehensive Test Of Phonological Processing—2” (CTOPP-2^45^. This is a promising result as Skibbe et al.^53^ report correlations of r = 0.25–0.57 between the verbal-response PA measures CTOPP-2 and the DIBELS (Dynamic Indicators of Basic Early Literacy Skills^68^, r = 0.57 between the CTOPP-2 and PELI (Preschool Early Literacy Indicators^69^, and a Pearson correlation of r = 0.58–0.65 between their own developed receptive PA measure and the CTOPP-2.

After establishing the convergent validity of the test, the ultimate goal of any PA assessment is to create a measure that can (a) identify children who are struggling to develop PA skills, (b) identify specific PA skills that could be targeted for training, and c) predict future reading performance so that educators can intervene before reading difficulties arise. The automated score reports from this online measure provide detailed information about the mastery of skills underlying each subtest as well as an overall percentile score that can be used to flag children who are at-risk of reading difficulties. Reporting mastery on individual subskills was supported by factor analysis, which revealed that First Sound Matching, Last Sound Matching and Deletion subtests represent a multi-dimensional framework (3 factors). Each subtest represented a correlated but distinct skill contributing to the latent PA construct. Therefore, this work supports the hypothesis that PA assessments do not necessarily reflect a single dimension^21–24^, and even within subtests (i.e., deletion) a hierarchical structure of skills^9,17^ should be considered (see Item difficulty assessment in Part 2). By establishing a good correlation between ROAR-PA and standardized reading scores such as the Woodcock-Johnson (r = 0.46 real words,r = 0.50 non-words^59^, and between ROAR-PA and ROAR-SWR (r = 0.53^51^, this assessment confirms the expected relationship between PA and reading skills (r = 0.46^12^, r = 0.41 (real words)—0.43 (non-words)^64^. A more detailed analysis of the relationship between the reading scores and subtests of the ROAR-PA showed that especially children with higher scores for the DEL subtest tend to have higher scores on the reading tasks.

Lastly, ROAR-PA was designed not just to work in a research environment or clinical setting, but to be available as a universal screener that could be used in schools. As a first examination of ROAR-PA in schools, we ran a study of first and second classrooms in California schools, substantiating the value of ROAR-PA in real-world, classroom settings. We found that the task could be administered simultaneously for a whole classroom and that it is predictive of current and future reading scores. Moreover, ROAR-PA in combination with ROAR-SWR achieved very high sensitivity for identifying students deemed “at risk” based on current standards of practice in the schools. However, specificity was lower indicating that the ROAR identified reading difficulties in students who performed adequately on the Fountas and Pinnell Benchmark Assessment. Understanding these discrepancies is an important challenge for future research to determine if (a) ROAR is sensitive to sub-skills that are missed by the courser measures like running records used by F&P versus (b) the automated ROAR measures are identifying some students who, in fact, perform well on measures with verbal responses. Distinguishing between these two alternative explanations will require further research, including longitudinal data collection, and using a battery of outcome measures.

Currently, ROAR-PA is a fixed order task with early stopping criteria implemented for stopping low performers earlier. Although this is already considered an adaptive task, for a future version of ROAR-PA we envision an even more efficient screening tool by implementing an Item Response Theory based computerized adaptive test. To maintain the strengths of the current PA task, we acknowledge the challenge to create an adaptive task that is sensitive to both the individual subtests and the composite score as a whole. Second, adding new items will allow us to assess PA skills at more regular intervals. And although PA is most valuable in early elementary school, adding more complex items could extend the utility of ROAR-PA to older children and adolescents with reading difficulties. In conclusion, we have shown through a series of design and validation studies that rapid, automated, online measures can lift the burden of resource-intensive, one-on-one assessment throughout childhood. These tools can facilitate developmental researchers to pursue larger, more diverse samples and catalyze discoveries into the mechanisms of learning that have applicability in clinics and classrooms.

## Methods

In the methods section, we delineate the progression and refinement of the ROAR-PA measure, tracing its development from its initial to its present iteration. Initially, we provide an overview of the task structure. Subsequently, we delve into the individual subtests and elucidate the rationale behind the tailored item development for both ROAR-PA version 1a and version 1b. Following this, we expound on participant demographics, recruitment procedures, and additional assessments administered to the participants. Finally, we detail the data collection process in Californian school districts, incorporating the changes implemented in the latest iteration of the ROAR-PA (Version 2).

### Receptive phonological awareness task

For the development of our receptive gamified online PA assessment, a one-interval, three-alternative forced choice task was created in the online study builder Lab.js^70^, converted to Javascript and uploaded to Pavlovia, an online experiment platform for hosting experiments^71^. This task, the ROAR-PA, was split into 5 subtests (25 or 24 items per subtest), with each subtest consisting of 2 or 3 blocks (divided by difficulty level). Each subtest started off with 3 training items with feedback. Training items had to be completed correctly before the task would continue to the test items. To engage children from pre-k to 8^th^ grade the task was embedded in a story where the child had to help a monkey and his four friends (rabbit, bear, otter and squirrel) collect their favorite foods. At the end of every trial some images of food would be displayed, at the end of every block a visualization of the collected food would be presented, and the character would provide encouragement (e.g., “*Great job! So many bananas! Let’s get a few more!”*). Every character would provide the instructions of their own subtest, guide throughout the task, motivate the participants to take short breaks between blocks, and introduce their next friend.

**Figure 8:**
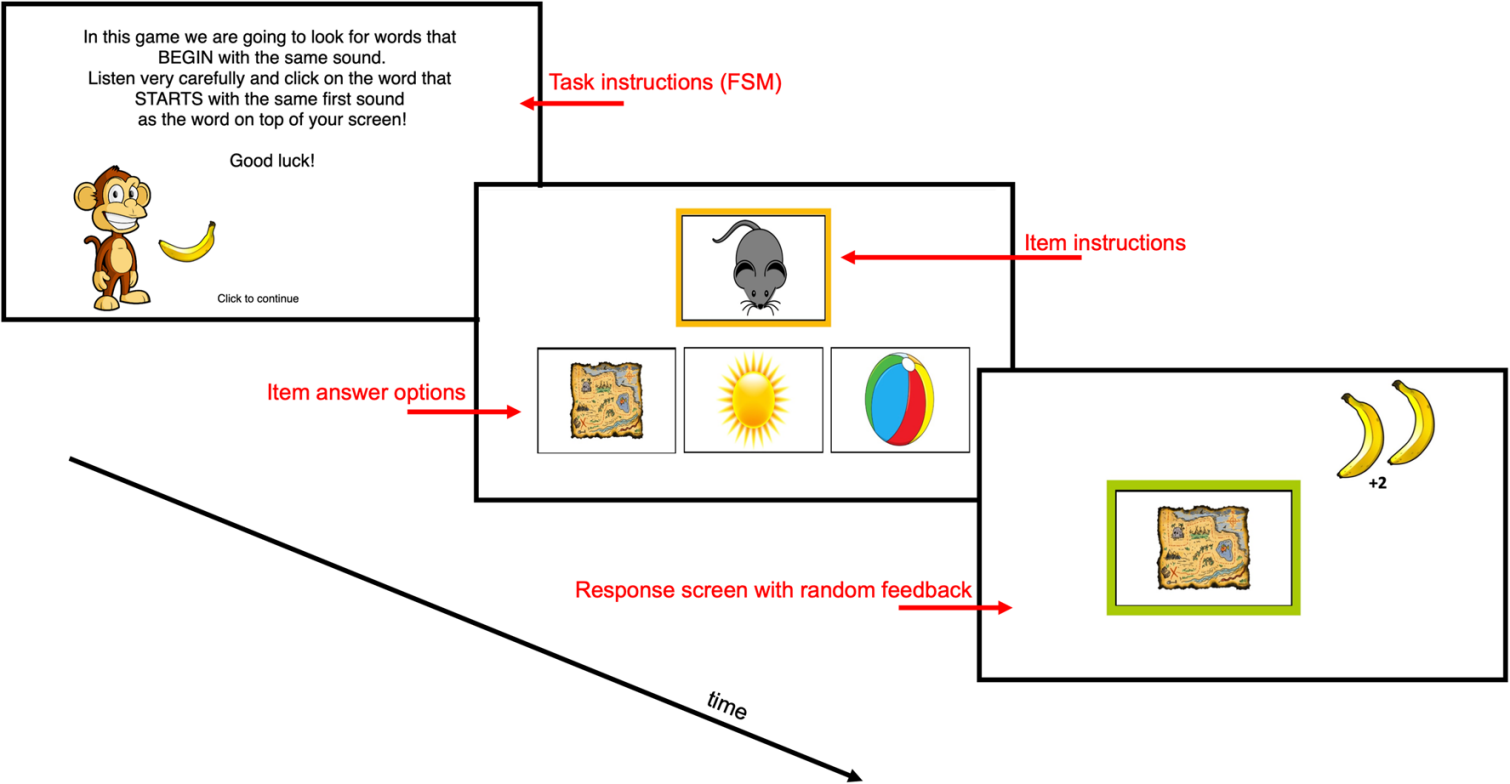
Visualization of experimental set-up. Every subtest (e.g., FSM) started with visual + auditory instructions, followed by some practice items with feedback. After the practice items 25 (or 24) items were presented in a 1I-3AFC task with random feedback (independent of answer, as motivation). The items were presented in semi-random order (within every block) and there were 2–3 sections per subtest. All items were presented visually (via clip-art images) + auditory.

Participants were instructed to to work on a computer (no table or phone) sitting at a desk. Sound had to be turned on and set to a comfortable level, based on the instructions of one of the characters. All game instructions were provided in both text and audio. For all trials of all subtests, one image accompanied by an audio fragment, recorded by a native English-speaking male, would provide the specific instruction of that trial (e.g., subtest FSM: “*Which picture starts with the same sound as dog?*”). This screen (Fig. 8) would be followed by the instruction image (top) plus three answer options (left, middle, right). All images would be verbalized (e.g., “dam” (target), “goat” (foil 1), “mop” (foil 2)) and the position of the images was randomized for each trial. For the BLE and DEL subtests the images were not verbalized as this could give away the correct answer. In contrast, participants were allowed to listen to the instruction phrase two times for these subtests. We did not implement this throughout the entire task to stay as consistent as possible with standardized PA tasks like the CTOPP-2 in which instructions are not repeated. Participants could pick a response by clicking the image, followed by a visualization of the response with a random number of food images as motivation (Fig. 8). All participants completed a total of 15 practice items and 123 trials. None of the trials, nor the breaks were limited in time, giving participants of all ages the opportunity to complete the tasks at their own pace.

### Subtests and stimuli (Version 1a)

The subtests were selected based on well-known standardized in-person PA tasks (e.g., PAT-2:NU^44^, CTOPP-2^45^, PPA^46^: First Sound Matching (FSM), Last Sound Matching (LSM), Rhyming (RHY), Blending (BLE) and Deletion (DEL). In FSM the participant had to find a word with the same first sound as a provided word,in LSM the last sound had to be recognized. In RHY, the participant had to find the word that rhymed with the provided word. In BLE parts of words had to be merged to one word. In DEL, a section of the word had to be omitted. FSM, LSM and RHY each consisted of 25 trials, divided into 2 blocks (16 and 9 items). The difference between blocks of these 3 subtests was finding the first sound (FSM), last sound (LSM), or word that rhymed (RHY) of a CVC word (difficulty level 1, 16 items) or a (C)CVC(C) word (difficulty level 2, 9 items). Thus, for the easier items (i.e., difficulty level 1) children had to identify an single phoneme (e.g., of FSM: Q: “Which picture starts with the same sound as pin?” A: “pup”), whereas for the more difficult items (i.e., difficulty level 2), children had to identify a consonant sound within a phoneme cluster (e.g., of FSM: Q: “Which picture starts with the same sound as clown?” A: “crab”). For FSM the three answer options were either the target (i.e., same first sound), a foil that started with the last sound of the provided word (Foil 1), or a foil with the same vowel (Foil 2). For LSM the same reasoning was made, but for the last sound of the word. For RHY the target word would rhyme, whereas Foil 1 would have the same vowel but not rhyme and Foil 2 would have the same first sound. BLE and DEL each consisted of 24 items, divided into 3 difficulty levels (i.e., syllable level, onset or rime level, phoneme level) with each 8 items. These difficulty levels were based on a suggested hierarchy within PA skills^9^^,^^17^^,^^25^. For example, for the subtest DEL an item of difficulty level 1 could be: Q: “What is lipstick without stick?” A: “lip”, for difficulty level 2: Q: “What is farm without ‘f’?” A: “arm”, and for difficulty level 3: Q: “What is snail without ‘n’?” A: “sail”. For both the BLE and DEL subtests, all additions and omissions led to lexical changes rather than morphological changes of the word structure.

An item was either scored as correct (i.e., target selected) or as incorrect (i.e., foil selected). No distinction was made in the scores based on which foil was selected.

### Subtests and stimuli (Version 1b)

Based on a first round of analysis (Item Response Theory analysis and correlation measures with the standardized CTOPP-2), the 3 most suitable subtests (FSM, LSM, and DEL) were selected to be included in version 1b of the ROAR-PA. The stimuli for this task were not adjusted (25, 25 and 24 items with 3 practice items per subtest).

### Participant recruitment and consent procedure

The parent or guardian of each participant provided informed consent under a protocol that was approved by the University of Washington’s (UW) Institutional Review Board and all methods were carried out in accordance with these guidelines. Each child participant was provided with assent via a conferencing call, previous to task assessment. Participants were recruited from one of two participant databases at UW: the University of Washington Reading & Dyslexia Research Program (http://ReadingAndDyslexia.com) or the UW Communication Participant Pool. A total of 143 participants (Age: 3.87–13.00, $\mu$ = 7.13, $\sigma$ = 1.89; Sex: 67 F, 76 M) completed the five subtests of ROAR-PA (Version 1a). The updated task (Version 1b) was completed by an additional group of 127 participants with mainly older children, resulting in 270 participants (Age: 3.87–14.92, $\mu$ = 9.12, $\sigma$= 2.71; Sex: 125 F, 145 M) completing ROAR-PA FSM, LSM and DEL.

### Assessment of other tasks

Of these 270 participants (Age: 3.87–14.92) from ROAR-PA_v.1, 266 completed the CTOPP-2 PA assessment via a video conferencing call. This task was assessed by a Speech Language Pathologist, as similar as possible to in-person testing. The Phonological Awareness Score was determined by 3 subtests: elision, blending, and phoneme isolation/ sound matching (depending on the age). In the same virtual session, we also assessed reading scores of 230 participants on the standardized Woodcock-Johnson Letter-Word-ID and Woodcock-Johnson Word-Attack task. Additionally, these participants completed the ROAR-SWR task via the web-browser.

### Data collected in school districts (Version 2)

After the analysis, a second version of the ROAR-PA was developed (ROAR-PA_v.2). We selected 3 subtests (FSM, LSM and DEL) with 19 items and 2 practice items per subtest. This resulted in a total of 57 trials + 6 practice trials. The task was broken up into 2 blocks per subtest. The items would now be presented in a fixed order, based on difficulty analysis, rather than being randomized within each block. To allow for efficient assessment, and to make the task accessible for both young and older children, a ceiling rule was implemented in this version of the ROAR-PA where the subtest will end if the participant gets either (a) 3 trials in a row incorrect, or (b) 4 out of 6 consecutive trials incorrect. This design is similar to the ceiling rule implemented in CTOPP-2. Similar to the original task, feedback would be given in the practice trials, but the actual trials only provided motivational rewards. For this validation, ROAR-PA_v.2 was administered to 50 1st and 2nd grade classrooms including 901 children in schools in the state of California. All study procedures were designed in collaboration with participating school districts, and ethical approval was obtained from the Stanford University institutional review board. At least two weeks prior to the administration of the ROAR, letters were sent home to all the families in the school districts describing the study and providing parents the opportunity to opt their children out of participation. The ROAR was not administered to children whose families opted out of the study. Children completed the ROAR-PA and ROAR-SWR tasks on Chromebooks in their classroom and were provided support in logging in but no assistance was given on the tasks. ROAR administration was done simultaneously for all the children in a classroom. Furthermore, reading levels were acquired via the Fountas and Pinnell Leveled Literacy Intervention^61^ through one-on-one assessments administered as standard of practice by the classroom teacher.

### Data analysis

All analysis code and data collected under the approval of the University of Washington’s Institutional Review Board are publicly available at: https://github.com/yeatmanlab/roar-pa-manuscript. Data collected in schools under the approval of the Stanford University institutional review board (Part 4: ROAR-PA_v2) are not publicly available due to privacy agreements. Data analysis was conducted in RStudio.

### Ethics approval

Parents of participants provided written informed consent under a protocol that was approved by the University of Washington’s Institutional Review Board for ROAR-PA_v.1. and ethical approval was obtained from the Stanford University institutional review board for ROAR-PA_v.2.

## Data Availability

All analysis code and data are publicly available at: https://github.com/yeatmanlab/roar-pa-manuscript. Data analysis was conducted in RStudio. Data collected in schools are not publicly available due to privacy agreements.
